# Next Generation Sequencing of Reactive Stroma and Residual Breast Cancer Cells in Tumor Bed after Neoadjuvant Chemotherapy

**DOI:** 10.3390/cancers14225609

**Published:** 2022-11-15

**Authors:** Zsuzsanna Varga, Ailsa Christiansen, Magdalena Lukamowicz-Rajska, Aashil A. Batavia, Adriana von Teichman, Peter Schraml, Holger Moch

**Affiliations:** Department of Pathology and Molecular Pathology, University Hospital Zurich, CH-8091 Zurich, Switzerland

**Keywords:** pathogenic driver mutations, chemotherapy, regressive tumor bed, breast cancer

## Abstract

**Simple Summary:**

Primary systemic or neoadjuvant chemotherapy of breast cancer has become a standard therapy option in locally advanced or triple negative or Her2 positive breast cancer. Neoadjuvant chemotherapy can result in complete pathological response without residual tumor cells (tumor bed) or partial response and non-response with different amounts of reactive stroma and residual tumor cells. In this study, we characterized the mutational status of residual breast cancer cells and reactive tumor stroma devoid of residual tumor cells in partial or non-responders using next generation sequencing. Pathogenic driver-mutations are exclusively restricted to residual carcinoma cells and are absent in reactive stroma independently from intrinsic breast cancer subtypes or tumor stage. These data suggest that the absence of pathogenic mutations in a tumor bed without residual tumor cells may have prognostic implications after neoadjuvant chemotherapy.

**Abstract:**

Primary systemic or neoadjuvant chemotherapy of breast cancer has become a standard therapy option in locally advanced or predefined intrinsic subtypes such as triple negative or Her2 positive breast cancer. Neoadjuvant chemotherapy can result in complete pathological response without residual tumor cells (tumor bed) or partial response and non-response with different amounts of reactive stroma and residual tumor cells. The interaction between therapy regimens and tumoral driver mutations have been extensively studied, although the reactive stroma of the tumor bed received less attention. In this study, we characterized the mutational status of residual breast cancer cells and reactive tumor stroma devoid of residual tumor cells in partial or non-responders using next generation sequencing. Twenty-one post-therapeutic breast surgical specimens after neoadjuvant chemotherapy underwent pathogenic driver-mutation screening using microdissected residual breast cancer cells and in reactive stroma adjacent to tumor bed areas. In reactive stroma, no mutations could be validated. In residual breast cancer cells, mutations were detected in sixteen of twenty-one cases (76%). In nine of these twenty-one cases (43%), pathogenic driver mutations (*PIK3CA, PTEN, TP53, FN1, PLAG1*) were identified. Pathogenic driver-mutations are exclusively restricted to residual carcinoma cells and are absent in reactive stroma independently from intrinsic breast cancer subtypes or tumor stage. These data suggest that the absence of pathogenic mutations in a tumor bed without residual tumor cells may have prognostic implications after neoadjuvant chemotherapy.

## 1. Introduction

Pre-operative chemotherapy or neoadjuvant chemotherapy (NAC) is a standard therapy option in locally advanced breast cancer for down staging the tumor or in cases with triple negative or Her2 positive intrinsic subtypes with expected high therapy response [1,2,3,4,5,6,7,8,9]. Pathological complete response (pCR) is the established surrogate endpoint for prediction of long-term clinical benefit, such as disease-free survival (DFS) and overall survival (OS) [1,2,3,4,5,6,7,8]. The effect of different therapy regimens, the interaction of drugs with carcinoma cells, and their response to therapy have been the subject of several studies for more than three decades [1,2,4,5,6,7,10]. The introduction of new therapy options, such as platinum containing or Her2 directed regimens in the neoadjuvant setting, contributed to improved OS and DFS [1,8]. With regard to pathological processing and reporting, major recent advances in the standardization of pathological and histopathological parameters have significantly influenced clinical decision making [3,11,12,13]. Whereas the presence of invasive residual carcinoma and the involved nodes have been the primary focus of several clinical and translational research studies, the stroma and its potential association with gene alterations after completing the pre-operative therapy have not yet been extensively studied [14,15,16,17,18,19,20,21].

Residual breast cancer cells are surrounded by the extracellular matrix and various cell types, such as fibroblasts, myofibroblasts, endothelial cells, and immune cells. There is evidence that there are molecular characteristics differentiating tumor-associated stroma from normal stroma and that the tumor-associated stroma contributes to cancer progression [19]. After primary systemic treatment, different patterns of breast cancer regression have been reported, including widely scattered tumor foci, a cellular tumor stroma with bizarre stromal cells or significant residual disease without clear gross tumor mass. However, data on genomic analyses of breast cancer tumor beds after neoadjuvant treatment are lacking. 

Therefore, we examined the tumor bed devoid of residual carcinoma cells in a partly-responder cohort following completion of neoadjuvant chemotherapy. We included patients with non-response and partial response (both referred as non-pCR) and performed a microdissection-based gene clustering analysis separately on both the tumor bed free of carcinoma cells, and the adjacent residual carcinoma cells. Our aim was to prove the hypothesis that a tumor bed without any residual tumor cells harbors no relevant driver or pathogenic mutations in contrast to residual carcinoma cells. 

## 2. Materials and Methods

### 2.1. Patient’s Cohort

Twenty-one female patients who underwent pre-operative systemic chemotherapy with consecutive breast surgery (mastectomy or local wide excision) were selected from the archived files of the Department of Pathology and Molecular Pathology, University Hospital Zurich, Switzerland between 2010 and 2013. The selection criteria were to preferentially include patients with non-response and partial response (both referred to as non-pCR) independently from the intrinsic or histological subtype with available clinical information as well as histological slides and paraffin blocks. The breast surgical specimens were available in formalin fixed paraffin embedded (FFPE) samples. Only breast cancer cases with sufficient DNA quality were included in the study, resulting in 4 triple negative, 4 Her2 positive, and 13 Her2 negative luminal breast cancer cases. The study was approved by the Cantonal Ethical Committee (KEK-2012-553). The cohort characteristics including postoperative tumor and nodal stage, and follow-up data (overall survival) are summarized in Table 1. 

### 2.2. Histological Characteristics in Breast Specimens after Neoadjuvant Treatment

Histological characteristics were defined according to the 2019 WHO classification of breast tumors and also using criteria from recommended reporting guidelines [11,12,22,23,24,25].

**Figure 1:**
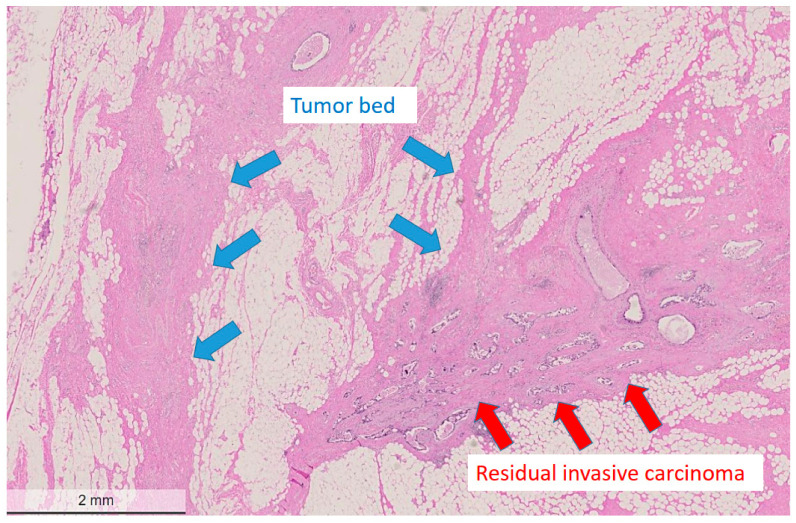
Low power view of a post-treatment breast surgical specimen after partial response to neoadjuvant chemotherapy. The tumor bed area adjacent to residual cancer cells is devoid of residual cancer cells and shows regressive fibrotic changes. The residual tumor cells display one residual measurable focus with a heterogeneous pattern with an estimated reduced cellularity of approximately 50%. Hematoxylin and eosin (H&E) stain, bar: 2 mm.

**Figure 2:**
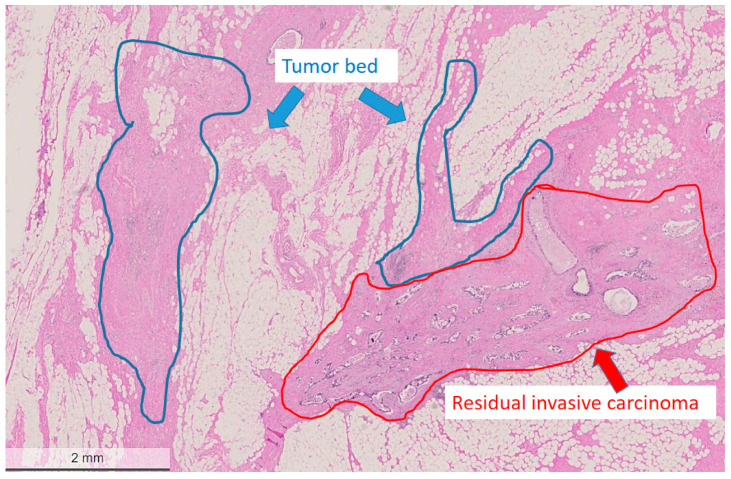
Areas marked for microdissection (tumor bed region circled with blue, residual tumor area circled with red). Hematoxylin and eosin (H&E) stain, bar: 2 mm.

**Figure 3:**
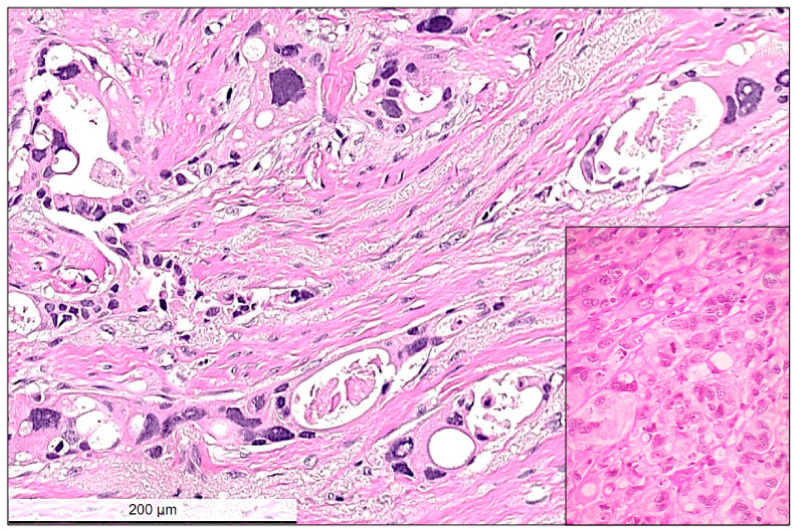
High power view of residual invasive carcinoma cells showing typical cytopathic post-chemotherapy changes, such as multinucleated bizarre cells, nuclear hyperchromasia, nuclear and cytoplasmic vacuolar inclusions. Between the residual carcinoma cells the stroma is loose, edematous corresponding to regression. Inset shows another example with no-response to chemotherapy. In this case, tumor cells remain tightly packed and the presence of atypical mitotic figures and hyperchromatic multinuclear cells with some intracytoplasmic vacuoles are visible. Hematoxylin and eosin (H&E) stain, bar: 200 micrometer.

**Pathological partial response (non-pCR):** Partial response was defined as a reduced amount of residual invasive carcinoma cells in less than 90% of the histologically verified tumor bed area (Figure 1 and Figure 2). Tumor cells in partial response were arranged in an irregular scattered pattern with a single cell or small clustered residual tumor cells with regressive stromal areas between the cellular areas. Residual cancer cellularity was semi-quantitatively assessed ranging from 1% to 100% per measurable residual tumor focus. Non-invasive residual tumor areas, such as ductal carcinoma *in situ* or lobular carcinoma in situ were not considered in partial response. The residual tumor cells had enlarged, hyperchromatic, multinuclear, and sometimes bizarre nuclei of different sizes often with intracytoplasmic or intranuclear vacuoles (Figure 3).

**Pathological non-response (non-pCR):** Non-response was defined as the presence of residual invasive carcinoma cells in more than 90% of the identifiable tumor bed area. In these cases, there was a thin rim of slightly regressive peripheral tumor bed area or complete lack of regression within the tumor cells. A previous biopsy site characterized by cystic macrophage reaction was often detectable within the tumor area. Cytopathic characteristics were similar to those observed in partial response or were completely lacking, and the tumor showed a similar cytomorphology as observed in the pre-operative core biopsy (Figure 3).

**Figure 4:**
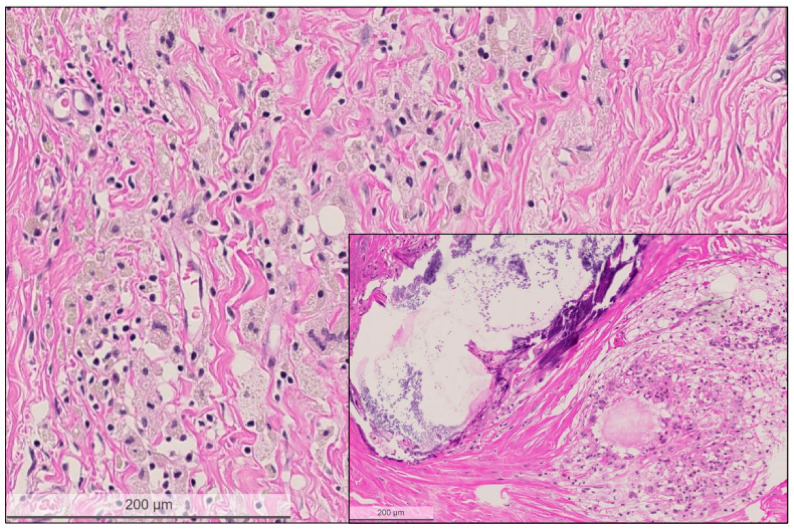
High power view of the regressive tumor bed devoid of residual carcinoma cells. The tumor bed area is characterized by loose collagenous stroma, macrophages lacking pre-existing tubulo-lobular units or invasive carcinoma. Inset shows another tumor bed area with large calcification and cystic macrophage reaction. Hematoxylin and eosin (H&E) stain, bar: 200 micrometer.

**Pathological complete response (pCR):** The tumor bed areas showing complete regression or a different regression pattern were evaluated both on low and high power magnification (Figure 1 and Figure 2). Complete regression was defined as complete lack of invasive carcinoma cells and also by the presence of a loose collagenous stroma with macrophages, foreign body reaction, cystic degeneration and biopsy sites, signs of previous bleedings (hemosiderin accumulation), and calcification lacking pre-existing tubulo-lobular units. In some cases, bizarre stromal cells were also visible in the tumor bed. In some surgical specimens, the gross identification of the tumor bed area at pathological complete response could be guided by localizing the pre-operative clip marks at the biopsy site (Figure 4). 

### 2.3. Next Generation Sequencing (NGS)

The areas of the tumor bed and the invasive residual carcinoma were micro-dissected from paraffin blocks guided by previously annotated corresponding areas on the hematoxylin and eosin (H&E) slides (as shown in Figure 2). The annotated areas on the H&E sections were manually matched with the surface of the corresponding paraffin block and the blocks were subsequently punched with a thin needle (0.6 mm in diameter). FFPE tissue biopsy punches from a total of 21 patients were taken from partially or non-responding breast cancer patients (non-pCR) following neoadjuvant therapy. Regions taken for analysis included both residual tumor and tumor bed as determined via histopathological assessment. While all care was taken during sampling to ensure only the tumor bed tissue was sampled, it is important to note that given the three-dimensional nature of the tissue block, it is possible that microscopic scattered tumor cells may have been sampled erroneously. All samples were subjected to NGS using the Ion AmpliSeq™ Comprehensive Cancer Panel comprising all exons of 409 known cancer genes (Life Technologies, Carlsbad, CA, USA). Input DNA for each run was 40 ng. Variant calling was performed using Ion Reporter (Thermo Fisher Scientific, Life Technologies, USA). Only variants detected with greater than 10% of reads and predicted to be damaging by SIFT and PolyPhen were included. All identified variants were confirmed in IGV and a selected number was verified by Sanger sequencing.

## 3. Results

**Figure 5:**
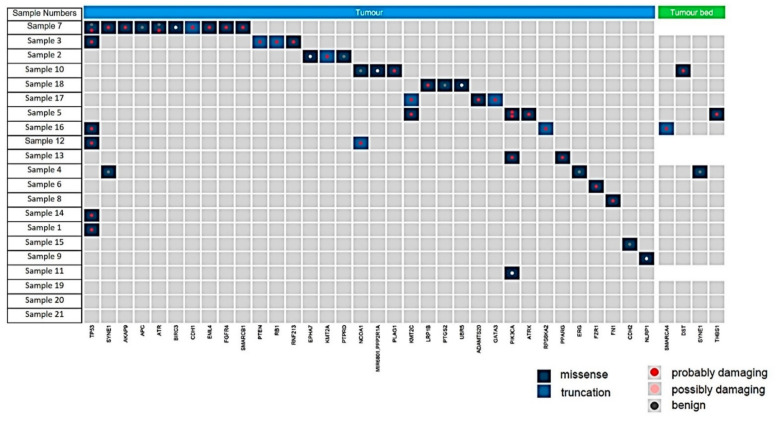
Summary of the mutations found in residual carcinoma (tumor, blue) and tumor bed (green). All mutations shown were detected at greater than 10% frequency of reads and are shown as missense (dark blue), truncating (light blue), and defined following the PolyPhen analysis as probably damaging (red dot), possibly damaging (pink dot) or benign (grey dot).

**Gene alterations in tumor bed:** Four mutations were identified in FFPE punches sampled from the tumor bed (4/21 patients) (Figure 5). In two patients (Nos. 5 and 10), single missense mutations were identified in *DST* and *THSB1* and were classed as “probably damaging” via PolyPhen analysis (Supplementary Table S1); however, subsequent analysis revealed that neither could be validated via Sanger sequencing. Another patient (No. 16) exhibited a possibly damaging truncating mutation in *SMARCA4*; however, this also could not be validated using Sanger sequencing. In addition, a benign missense mutation in *SYNE1* was identified in the tumor bed of another patient (No. 4). No other mutations were detected in the remaining 17 patients analyzed.

**Gene alterations in residual invasive carcinoma:** Mutations were detected in 16 of 21 residual carcinomas (76%), with some carcinomas carrying multiple genetic lesions. Pathogenic mutations, such as *PIK3CA, PTEN, TP53, FN1, PLAG1* were detectable in 9 of 21 residual cancers (50%). Notably, one tumor harbored 12 mutations in 10 genes. Details of the detected mutations are listed in Supplementary Table S2 and shown in Figure 5.

### 3.1. Tumor Infiltrating Lymphocytes (TILs)

The presence of TILs could be semi-quantitatively assessed on conventional routine hematoxylin and eosin (H&E) stains in 16 of 21 cases with a three-tiered scoring for the presence of TILs (0, +, ++, ++). Extensive TILs were present in 3 of 16 cases, scattered or lacking TILs were evidenced in 13 further cases. There was no association between TILs and the presence of mutational status, pathological response or follow-up (details are listed in Table 1).

### 3.2. PD-L1 Status

PD-L1 status was immunohistochemically assessed using the SP142 antibody in 16 of 21 cases. The reactions were carried out on the Ventana Benchmark with the Ventana SP-142 ready-to-use antibody according to the manufacturer’s instructions including scoring. Intratumoral immune cell (IC) positivity of at least 1% was considered as positive. Moreover, 12 cases were negative, and 4 cases showed positive IC scores. There was no association between IC scores and the presence of mutational status or follow-up (details are listed in Table 1).

### 3.3. Statistical Analysis

The mutation detection rate between the different intrinsic subtypes and pathological response rate groups underwent statistical comparison using Fisher’s exact probability test. The differences in the mutation detection rate among intrinsic subtypes were statistically not significant (*p* = 0.634). When analyzing the presence of TILs and mutation rate in the partial responder and non-responder, the statistical difference was also not significant (*p* = 0.168).

## 4. Discussion

Pre-operative chemotherapy or neoadjuvant chemotherapy is a standard therapy option in locally advanced breast cancer and in cancers with triple negative or Her2 positive intrinsic subtypes [1,2,3,4,5,6,7,8]. As proven in several previous studies and recommended by international guidelines, surrogate endpoint of a pre-operative systemic therapy is the pathological complete response for prediction of long-term disease-free survival (DFS) and overall survival (OS) [1,2,3,4,5,6,7,8]. 

In this study, we could show that driver or pathogenic mutations are completely absent in the adjacent regressive tumor bed and are essentially restricted to residual carcinoma cells in non-pCR independent from intrinsic subtypes or tumor stage. The absence of any pathogenic mutations within the tumor bed devoid of tumor cells may be considered as a molecular evidence that supports the complete pathological response as a favorable prognostic factor. 

We are not aware of any previous study that comprehensively analyzed genomic alterations in the tumor bed devoid of residual carcinoma cells after pre-operative systemic therapy.

Residual cancer cellularity was semi-quantitatively assessed ranging from 1% to 100% per measurable residual tumor focus. This may be a reason why in five cases (with low percentage of tumor cells) no mutations have been detected. It is important to note that given the three-dimensional nature of the tissue block, it is possible that microscopic scattered tumor cells may have been sampled erroneously. However, the absence of detectable mutations in the tumor beds indicates a reliable strategy to investigate cells in these areas.

The pathological classification system of the residual cancer burden (RCB) for reporting residual disease and therapy response has been widely used and recommended by pathological and clinical guidelines [3,8,11,12,24]. Cytopathic changes (multinuclear bizarre cells, nuclear and cytoplasmic vacuolization, heterogeneous cellularity and multifocality) along with the largest measurable residual cancer manifestation, as well as the presence of regressive changes in the tumor bed (macrophage reaction, calcification cysts, loose fibrosis) are established histological criteria in pathology reports [3,8,11,12,24,26].

Morphological cellular and stromal changes with prognostic relevance have not been extensively explored in the literature [3,12]. Zombori *et* Cserni reported that multinucleated residual tumor giant cells occur in approximately 18% of cases following NAC almost exclusively in patients who received regimens containing taxanes. Moreover, this study observed that there was no correlation between intrinsic subtypes and the presence of regressive stromal changes [12]. Hasebe et al. described an association between the presence of atypical benign stromal cells and recurrent disease after NAC [3]. Furthermore, a higher tumor-stroma ratio (low stromal presence) in pre-operative biopsies has been associated with a higher pCR rate after NAC pointing to the prognostic relevance of the accompanying stroma in neoadjuvant treatment [27].

Very few studies have investigated protein expression in the tumor bed following NAC [14,15,16,18,20,21,28,29]. In recent years however, tumor infiltrating lymphocytes (TILs) post-NAC have gathered more attention. Increased levels of tumor associated M2 macrophages (M2 TAM) in the pre-operative biopsy tissues, such as CD68 or CD163 positive macrophages, have been associated with reduced responses to therapy and more frequent non-pCR results [28,29]. The anatomical intra-tumoral immune cell localizations (stroma vs. infiltration front vs. peritumoral location) have been shown to impact both partial response, stable disease, and metastatic properties [18,29,30]. The role of tumor infiltrating lymphocytes (TILs) and their association with PD-L1 expression and pCR after NAC have been the subject of several studies and are still being debated in clinical consensus treatment recommendations [1,14,15]. In the pre-operative setting, higher baseline TILs and PD-L1 have been shown to positively impact pCR [1,14,15]. While a trend toward a reduction in stromal TIL counts in the post-NAC specimens of patients with pCR has been reported in several studies [1,14,15]. High baseline TILs together with low stromal clusterin expression were associated with a higher likelihood of achieving a complete pathological response, which was independent from other clinico-pathological parameters, such as age, tumor/nodal stage or histological subtype [21,30]. Concerning stromal expression of epithelial mesenchymal transition proteins (EMT), Riemenschnitter et al. reported that strong stromal Sox9 expression after NAC in breast cancer was associated with shortened overall survival, and stromal slug expression was significantly reduced in post-NAC surgical specimens [16]. In our cohort, we could not confirm any association between PD-L1 status, TILs and response to therapy, follow-up or mutational status.

Data on stromal gene alterations and stroma-related gene signature in the neoadjuvant setting is also not well understood [17,19,31]. The reciprocal role of cancer associated fibroblasts (CAF) expressing cognate receptors and cancer cells expressing platelet-derived growth factor (PDGF)-CC has been linked to the pathomechanism to explain how conversion of intrinsic subtype from basal-like to luminal cancer can develop after administration of chemotherapy [17]. An altered gene signature in stroma (the so-called HER2STROMA gene signature set) has been identified in patients who failed to respond to HER2 directed therapy, pointing to a further possible mechanism in trastuzumab resistance, which in this cohort was independent from the presence of stromal TILs [19]. The EORTC 10994/BIG 00-01 trial investigated the role of stromal gene signature alterations in post-therapy surgical samples in patients who experienced chemotherapy failure [31]. This trial shows that in micro-dissected tumor samples, stromal gene signature alterations were found in the reactive stromal component pointing again to a possible previously undescribed pathomechanism of therapy resistance [31].

## 5. Conclusions

In summary, our data show a potential link between the clinical endpoint of pathological complete response and the lack of genetic alterations in the tumor bed devoid of carcinoma cells in patients receiving pre-operative systemic chemotherapy. We could demonstrate drivers or pathogenic mutations which are often seen in high-risk breast cancer only in the residual carcinoma cells but not in the regressively changed tumor bed areas. These results, which were also independent of intrinsic or histological subtype, age or tumor stage may add additional molecular information to the clinical definition of complete pathological response after neoadjuvant chemotherapy in breast cancer. Under-recognized molecular changes in the tumor-free regression zone after neoadjuvant chemotherapy should be included in the interaction between therapy regimens and response to therapy effect. However, more confirmatory studies are necessary to verify the consistency of the results in this study.

## Figures and Tables

**Table 1:** Clinicopathological characteristics of the cohort along with the results of the next generation sequencing analyses. abbreviations: Response: CR Complete responder (only DCIS evidence in case 1), NR Non-responder, PR Partial responder, Status: ER Estrogen Receptor, PR: progesterone Receptor, TILs: tumor infiltrating lymphocytes.

Patient’s Number	Age at Diagnosis [Years]	cT,cN	ypT	ypN	ER/PR /Her2 Status	PD-L1 Status	TILs	Response	Follow-Up [Years]	Mutations in Tumour Cells	Mutations in Tumour Bed
1	43	T2/N1	pTis	N1	triple negative	NA	NA	CR	2 years dead	TP53	no mutations
2	74	T4b/N1/M1	pT4d	N2	ERpos /PRpos /Her2neg	neg	+	NR	1 year dead	EPHA7, KMT2A	no mutations
3	50	T2/N1	pT2	N0	triple negative	neg	+	NR	13 years alive	PTEN, TP53, RNF213, RB1	no mutations
4	62	T3/N1	pT3	N0	ERpos /PRneg /Her2 neg	pos IC 2%	+	NR	11 years dead	no mutations	no mutations
5	40	T2/N1/M1	pT1c	-	ERpos /PRpos /Her2neg	neg	0	NR	10 years dead	PIK3CA, KMT2A, ATRX	no mutations
6	45	T4/N0	pT2	N0	ERpos /PRpos /Her2pos	neg	0	NR	11 years alive	FZR1	no mutations
7	76	T2/N1	pT2	N1	ERpos /PRpos /Her2neg	pos IC 5%	+++	NR	11 years dead	EML4, ATR, FGFR4, SYBE1, AKAP9, BIRC3, CDH1, TP53	no mutations
8	45	T2/N0	pT1b	-	ERneg /PRneg /Her2pos	neg	0	NR	8 years dead	FN1	no mutations
9	56	T2/N1	-	N1	triple negative	pos IC 2%	+++	NR	6 years dead	NLRP1	no mutations
10	82	T3/N2	pT3	N2	triple negative	pos IC 5%	+++	NR	9 years dead	PLAG1, MR6801, PP2R1A	no mutations
11	38	T2(m)/N2	pT1c	N2	ERpos /PRpos /Her2neg	neg	0	NR	12 years alive	PIK3CA	no mutations
12	54	T2/N1	pT2	N1	ERpos /PRpos /Her2neg	NA	NA	NR	lost to follow-up	TP53, NCOA1	no mutations
13	67	T1/N0	pT2	N0	ERpos /PRpos /Her2neg	neg	0	NR	9 years alive	PPARG. PIK3CA	no mutations
19	50	T2/N0	pT2	N0	ERpos /PRpos /Her2pos	NA	NA	NR	lost to follow-up	no mutations	no mutations
20	39	T3/N1/M1	pT3	N1	ERpos /PRpos /Her2neg	NA	NA	NR	4 years dead	no mutations	no mutations
21	66	T2/N1	pT3	N0	ERpos /PRpos /Her2neg	NA	NA	NR	12 years alive	no mutations	no mutations
14	68	T2/N1	pT2	N1	ERpos /PRpos /Her2pos	neg	++	PR	7 years dead	TP53	no mutations
15	43	T3/N1	pT3	N1	ERpos /PRpos /Her2neg	neg	+	PR	9 years alive	no mutations	no mutations
16	43	T4/N1/M1	pT3	N2	ERpos /PRpos /Her2neg	neg	+	PR	4 years dead	RPS6KA2	no mutations
17	72	T2/N0	pT2	N0	ERpos /PRpos /Her2neg	neg	+	PR	lost to follow-up	KMT2C, GATA3, ADAMTS20	no mutations
18	37	T1(m)/N1	pT1a	N1	ERpos /PRpos /Her2neg	neg	0	PR	lost to follow-up	LRP1B, UBR5	no mutations

## Data Availability

All data are available upon reasonable request from the corresponding author.

## Supplementary Materials

The following supporting information can be downloaded at: https://www.mdpi.com/article/10.3390/cancers14225609/s1. Table S1: Detailed tumor bed tissue mutations identified via ION torrent analysis, none of which could be validated via subsequent Sanger sequencing of the tumor bed FFPE tissue punches. Table S2: Detailed mutations detected in residual carcinoma FFPE tissue punches.
