# Supplementary material for: Next Generation Sequencing of Reactive Stroma and Residual Breast Cancer Cells in Tumor Bed after Neoadjuvant Chemotherapy
Source: Cancers (Basel). 2022 Nov 15;14(22):5609. doi: 10.3390/cancers14225609 (PMC9688915; doi:10.3390/cancers14225609)
Supplement: Supplementary file 1 [file cancers-14-05609-s001.zip › Supplementary Table S1 final version 23.09.2022.pdf]

Supplementary Table S1: Detailed tumor bed tissue mutations identified via ION torrent analysis, none of which could be validated via subsequent Sanger Sequencing of the tumor bed FFPE tissue punches.

Patient ID	Chromosomal Position	Genes	Reference	Observed allele	% Frequency	Coding	Amino Acid Change	Variant Effect
6								no mutations
8								no mutations
14								no mutations
1								no mutations
15								no mutations
16	chr19:11097620	SMARCA4	G	GC	36.42	c.801_802insC	p.Val268fs	truncation
9								no mutations
10	chr6:56341107	DST,RNU6-71P	T	A	20.08	c.15347A>T	p.Gln5116Leu	missense
18								no mutations
11								no TB data
12								no TB data
2								no mutations
7								no TB data
13								no TB data
17								no mutations
3								no mutations
4	chr6:152590401	SYNE1	C	G	17.11	c.18594G>C	p.Glu6198Asp	missense
5	chr15:39884882	THBS1	G	T	31.21	NM_003246.2	c.2646G>T	missense
21								no mutations
19								no mutations
20								no mutations
