# Supplementary material for: Next Generation Sequencing of Reactive Stroma and Residual Breast Cancer Cells in Tumor Bed after Neoadjuvant Chemotherapy
Source: Cancers (Basel). 2022 Nov 15;14(22):5609. doi: 10.3390/cancers14225609 (PMC9688915; doi:10.3390/cancers14225609)
Supplement: Supplementary file 1 [file cancers-14-05609-s001.zip › Supplementary Table S2 final version 23.09.2022.pdf]

Table S2: Table details mutations detected in residual carcinoma FFPE tissue punches.

Patient ID	Chromosomal Position	Genes	Reference	Observed allele	% Frequency	Coding	Amino Acid Change	Variant Effect
6	chr19:3532070	FZR1	G	A	10.43	c.985G>A	p.Ala329Thr	missense
8	chr2:216264003	FN1	G	A	23.81	c.3325C>T	p.Pro1109Ser	missense
14	chr17:7574017	TP53	C	T	16.36	c.1010G>A	p.Arg337His	missense
1	chr17:7578259	TP53	A	C	15.03	c.590T>G	p.Val197Gly	missense
15	chr18:25570167	CDH2	C	A	11.78	c.1492G>T	p.Ala498Ser	missense
16	chr6:167275623	RPS6KA2	C	CT	10.91	c.35_36insA	p.Ile13fs	truncation
	chr17:7578479	TP53	GT	AT	12.55	c.451C>T	p.Pro151Ser	missense
9	chr17:5462417	NLRP1	C	A	16.53	c.1599G>T	p.Gln533His	missense
10	chr19:52725418	MIR6801,PPP2R1A	G	C	14.17	c.1585G>C	p.Ala529Pro	missense
	chr2:24965004	NCOA1	A	T	17.44	c.3655A>T	p.Thr1219Ser	missense
	chr8:57080049	PLAG1	G	T	12.27	c.256C>A	p.His86Asn	missense
18	chr2:141267539	LRP1B	C	T	20.35	c.8356G>A	p.Asp2786Asn	missense
	chr1:186643892	PTGS2	C	G	12.23	c.1408G>C	p.Glu470Gln	missense
	chr8:103300458	UBR5	G	A	23.7	c.4750C>T	p.His1584Tyr	missense
11	chr3:178952085	PIK3CA	A	G	11.75	c.3140A>G	p.His1047Arg	missense
12	chr2:24914463	NCOA1	C	T	32.55	c.646C>T	p.Gln216Ter	truncation
	chr17:7578526	TP53	CA	AA	62.27	c.404G>T	p.Cys135Phe	missense
2	chr6:94067998	EPHA7	C	T	66.3	c.964G>A	p.Asp322Asn	missense
	chr11:118352786	KMT2A	C	T	11.94	c.3991C>T	p.Gln1331Ter	truncation
	chr9:8485319	PTPRD	C	A	64.45	c.3061G>T	p.Ala1021Ser	missense
7	chr7:91641851	AKAP9	C	G	15.26	c.3427C>G	p.Leu1143Val	missense
	chr5:112137018	APC	G	A	12.95	c.772G>A	p.Glu258Lys	missense
	chr3:142180920	ATR	C	G	21.32	c.7054G>C	p.Asp2352His	missense
	chr3:142231122	ATR	C	T	12.61	c.4832G>A	p.Arg1611Lys	missense
	chr11:102206912	BIRC3	C	G	12.57	c.1540C>G	p.Leu514Val	missense
	chr16:68849652	CDH1	C	T	10.45	c.1555C>T	p.Gln519Ter	truncation
	chr2:42508091	EML4	G	C	13.56	c.769G>C	p.Glu257Gln	missense
	chr5:176519496	FGFR4	A	G	30.84	c.902A>G	p.Tyr301Cys	missense
	chr22:24167549	SMARCB1	C	G	10.02	c.933C>G	p.Ile311Met	missense
	chr6:152651974	SYNE1	C	T	16.4	c.13846G>A	p.Glu4616Lys	missense
	chr17:7577085	TP53	C	G	36.7	c.853G>C	p.Glu285Gln	missense
	chr17:7577506	TP53	C	A	21.59	c.775G>T	p.Asp259Tyr	missense
13	chr3:178936082	PIK3CA	G	A	20.92	c.1624G>A	p.Glu542Lys	missense
	chr3:12447480	PPARG	G	C	22.28	c.719G>C	p.Arg240Pro	missense
17	chr12:43944872	ADAMTS20	G	T	47.91	c.293C>A	p.Ser98Tyr	missense
	chr10:8115874	GATA3	C	CG	31.57	c.1224_1225insG	p.Pro409fs	truncation
	chr7:151879307	KMT2C	G	A	29.68	c.5638C>T	p.Gln1880Ter	truncation
	chr10:89717769	PTEN	TAA	TAT	16.34	c.796A>T	p.Lys266Ter	truncation

3	chr13:48942673	RB1	CAG	C	10.18	c.1064_1065delGA	p.Arg355fs	truncation
	chr17:78319808	RNF213	C	A	11.59	c.7673C>A	p.Ser2558Tyr	missense
	chr17:7577538	TP53	CG	TG	20.83	c.743G>A	p.Arg248Gln	missense
4	chr21:39817472	ERG	T	A	23.87	c.91A>T	p.Thr31Ser	missense
	chr6:152590401	SYNE1	C	G	32.1	c.18594G>C	p.Glu6198Asp	missense
5	chrX:76949378	ATRX	T	C	14.06	c.419A>G	p.Asp140Gly	missense
	chr7:151880211	KMT2C	G	A	14.82	c.5113C>T	p.Arg1705Cys	missense
	chr3:178936074	PIK3CA	C	G	14.82	c.1616C>G	p.Pro539Arg	missense
	chr3:178936095	PIK3CA	A	C	13.64	c.1637A>C	p.Gln546Pro	missense
21	no mutations							no mutations
19	no mutations							no mutations
20	no mutations							no mutations
